# Basis and Design of a Randomized Clinical Trial to Evaluate the Effect of Jinlida Granules on Metabolic Syndrome in Patients With Abnormal Glucose Metabolism

**DOI:** 10.3389/fendo.2020.00415

**Published:** 2020-06-25

**Authors:** De Jin, Lili Hou, Shuolong Han, Liping Chang, Huailin Gao, Yiru Zhao, Shenghui Zhao, Xuedong An, Guangyao Song, Chunli Piao, Fengmei Lian, Tong Xiao-lin, Zhenhua Jia

**Affiliations:** ^1^Department of Endocrinology, Guang'anmen Hospital of China Academy of Chinese Medical Science, Beijing, China; ^2^Department of Cardiovascularology, Key Laboratory of State Administration of TCM (Cardio-Cerebral Vessel Collateral Disease), Shijiazhuang, China; ^3^Department of Cardiovascularology, National Key Laboratory of Collateral Disease Research and Innovative Chinese Medicine, Shijiazhuang, China; ^4^Department of Cardiovascularology, Key Disciplines of State Administration of TCM for Collateral Disease, Shijiazhuang, China; ^5^National Administration of Traditional Chinese Medicine (TCM) Regional TCM Diagnosis and Treatment Center (Cardiovascular Disease), Shijiazhuang, China; ^6^Department of Endocrinology, Heibei Yiling Hospital, Shijiazhuang, China; ^7^Guangzhou University of Traditional Chinese Medicine, Shenzhen Hospital, Guangzhou University of Chinese Medicine, Shenzhen, China

**Keywords:** Jinlida granules, Chinese medicine, metabolic syndrome, abnormal glucose metabolism, clinical trial

## Abstract

**Background:** Metabolic syndrome (MS) is a powerful risk factor for cardiovascular and cerebrovascular diseases. Although lifestyle intervention reduces several of the symptoms of the syndrome and cardiovascular risks, the lifestyle intervention that yields the benefits is restrictive. Jinlida is a Chinese patent medicine that has shown activity in type 2 diabetes, which has been approved in China. Preclinical studies in Jinlida granules support an improved role of abnormal glucose and lipids metabolism as well as reducing weight. Here, we describe the protocol of an ongoing clinical trial investigating a new therapy for metabolic syndrome in patients with abnormal glucose metabolism.

**Methods:** This study will enroll 880 subjects (aged 18–70 years) who have metabolic syndromes with abnormal glucose metabolism. All the participants in a double-blind, parallel, randomized, placebo-controlled trial, will receive Jinlida or placebo, orally, 9 g/time, three times daily for 2–4 years period on the basis of lifestyle intervention. The primary outcome measure (Incidence of type 2 diabetes) will be assessed during intervention cycles. Adverse events were monitored. All statistical tests will be performed using a two-sided test, and a *p* ≤ 0.05 (two-sided test) will be considered to be statistically significant results.

**Discussion:** Results from this study will provide evidence on whether incorporating oral Jinlida granules treatment into lifestyle intervention can delay or inhibit the development of diabetes mellitus in metabolic syndrome subjects with abnormal glucose metabolism.

**Clinical trial registration:** Registered at http://www.chictr.org.cn/enIndex.aspx. Trial registration number: ChiCTR1900023241.

## Background

The metabolic syndrome is a cluster of the most dangerous heart attack risk factors: diabetes and prediabetes, abdominal obesity, high cholesterol, and high blood pressure. The total risk of death was increased by a factor of 1.5 compared with non-metabolic syndrome (1). A cohort study from China demonstrated that the incidence of atherosclerosis cardiovascular disease (ASCVD) in subjects with metabolic syndrome was 3.12 times greater than those without metabolic syndrome (2). Additionally, as the global urbanization process accelerates, the population of metabolic syndrome further expands. According to data published by the International Diabetes Federation (IDF), a quarter of the world's population suffered from metabolic syndrome (3, 4). Importantly, especially in MS with diabetes and prediabetes, it contributed markedly to the development of ASCVD (5, 6).

Moreover, some studies have shown that 33% with impaired fasting glucose (IFG) and 71% with impaired glucose tolerance (IGT) also suffered from metabolic syndrome (7), which magnify the risk of ASCVD. As levels of blood glucose rises, even if the diagnostic criteria for diabetes (e.g., IFG, IGT) is not reached, the risk of ASCVD is still significantly increased (8–10). IGT is more common than IFG. Correspondingly, the risk of developing diabetes and cardiovascular disease in the IGT population is significantly higher than that of the IFG population (11). Daqing study demonstrated that ~90% of IGT subjects developed type 2 diabetes within 20 years, and half of them had at least one myocardial infarction or stroke event (12).

Urgent action to treat IGT in metabolic syndrome is especially necessary. Currently, the lifestyle intervention is widely recommended in clinical practice. Although active lifestyle intervention has been shown to be an effective strategy, the lifestyle intervention that yields the benefits is still restrictive and difficult to achieve in all the patients (13). Jinlida is a multitargeted antimetabolite that has shown activity in type 2 diabetes, which has been approved in China and consists of danshensu sodium salt, puerarin, salvianolic acid B, epimedin B, epimedin C, lcariin, and ginsenosides Rb1, Rc, and Rb2. The Jinlida, in one batch number, was manufactured by Shijiazhuang Yiling Pharmaceutical Co. (Shijiazhuang, China). Findings from pharmacological researches on Jinlida demonstrated its function protecting islet β-cell (14), anti-oxidative stress (15, 16), regulating hormones related with blood glucose (17), and protecting vascular endothelial cells (18). Additionally, Jinlida could reduce insulin resistance by regulating lipid metabolism (19, 20), promoting skeletal muscle gene and protein expression (21, 22), all of which play a key role in anti-metabolic disorders.

Our research team will implement a randomized, double-blind, placebo-controlled, and multi-center clinical trial to investigate the efficacy and safety of intervention with Jinlida granules in metabolic syndrome with abnormal glucose metabolism. This work describes the methodology and specific details underlying the study.

## Methods/Design

### Study objectives

In this study setting, the metabolic syndrome was defined as abdominal obesity, prediabetes (Impaired Glucose Tolerance), high cholesterol, and high blood pressure, not including diabetes. The aim of this study is to evaluate the effectiveness of Jinlida in delaying or inhibiting the development of diabetes mellitus in metabolic syndrome subjects with impaired glucose tolerance. We hypothesize that general lifestyle intervention + Jinlida granule is more effective than placebo + general lifestyle intervention in delaying or inhibiting the development of diabetes mellitus in metabolic syndrome subjects with impaired glucose tolerance. If successful, this work will provide clinical evidence for high-risk factors requiring intervention to prevent arteriosclerosis and inhibit the development of the cardiovascular pathology.

A total of 880 patients will be enrolled in this study over the course of 2 years. Recruitment takes place at an out-patient clinical institution. The principal investigators or investigators inform the patients (orally and in writing) about benefits and risks of participating in the study and of the clinical assessments and randomized allocation of treatment. Patients have to provide written consent before enrolling in the study. Patients may withdraw their consent to participate at any time without providing a reason. There is no financial compensation for the subjects. However, all clinical evaluations and study medication have no cost for the patients.

### Study Design

**Figure 1:**
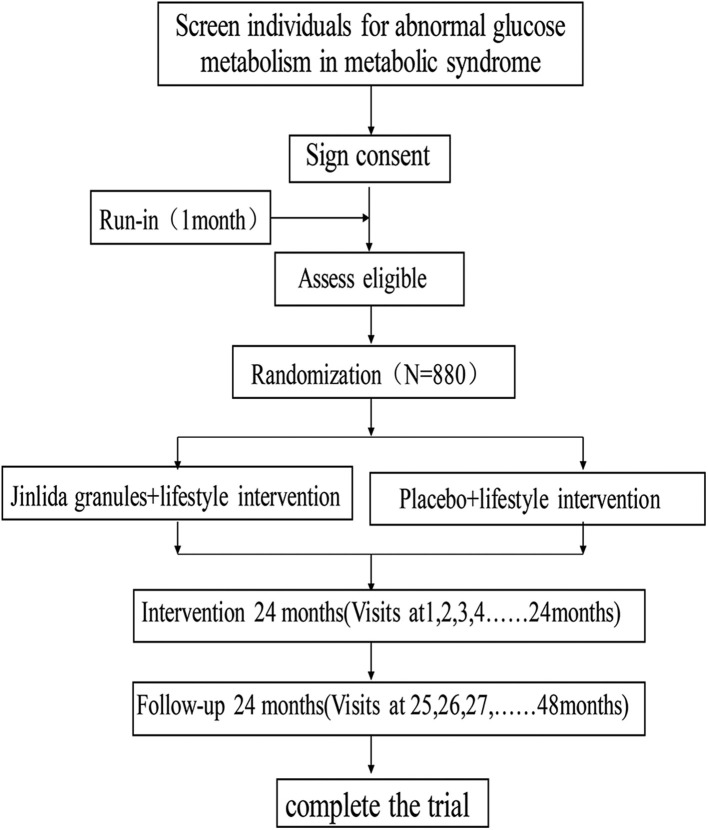
The schedule of enrollment, interventions and assessments.

This study is designed as a randomized, double-blind, placebo-controlled, multicenter clinical trial with a ratio of 1:1, which includes 22 hospitals (Research Cooperation Center). The trial was registered at the Chinese Clinical Trial Registry (ChiCTR, www.chictr.org.cn); ID: ChiCTR1900023241, and any important changes in the protocol will be reflected there. Figure 1 shows the study flow.

### Setting and Participants

This study will be conducted at 22 Collaborative Research Centers in China. Recruitment will be by advertisement in local free papers and by posters displayed in 22 Collaborative Research Centers as well as we disseminate relevant recruitment information in the community. The recruitment began in August 2019, and it will be completed within 2 years. All the participants will receive oral and written information regarding the study by a doctor involved in this work.

### Eligibility Criteria

#### Inclusion Criteria

1. Subjects are between 18 and 70 years of age;
2. After the run-in period, subjects should meet the diagnostic criteria for metabolic syndrome (3, 6):

- Indispensable indicators:
  Abdominal obesity (central obesity): waist circumference in male ≥ 90 cm, female ≥ 85 cm;
- Other indicators:
  At least two of the following four indicators can diagnose metabolic syndrome:
  ① Hyperglycemia: fasting blood glucose (FBG) ≥ 6.1 or 2 h after glucose load (2HPG) ≥ 7.8 mmol/L and/or have been diagnosed as diabetes and received treatments.
  ② Hypertension: blood pressure ≥ 130/85 mmHg and/or have been confirmed to be hypertension and received related treatments.
  ③ Fasting TG ≥ 1.70 mmol/L.
  ④ Fasting HDL-C <1.04 mmol/L.

3. After the run-in period, subjects should meet the diagnostic criteria for impaired glucose tolerance:
(a) Impaired fasting glucose (IGT): FBG < 7.0 mmol/L and 2HPG: 7.8–11.1 mmol/L.

#### Exclusion Criteria

1. Subjects who have used hypoglycemic drugs in the past 3 months;
2. Hyperthyroidism or hypothyroidism caused by endocrine disorders;
3. Uncontrollable hypertension/hypotension: systolic blood pressure ≥200 mmHg or diastolic blood pressure ≥110 mmHg; or systolic blood pressure ≤ 90 mmHg, or diastolic blood pressure ≤ 60 mmHg;
4. Comorbidities such as various acute infections, or severe infections, severe anemia, and neutropenia;
5. Major disease comorbidities, such as active or untreated malignant tumors, or clinical remission of malignant tumors <5 years ago;
6. Natural heart disease, such as congenital heart disease, rheumatic heart disease, hypertrophic or dilated cardiomyopathy, New York Heart Association (NYHA) cardiac function classification > III level;
7. Subjects who had any of the following conditions within the past 6 months: coronary intervention (e.g., CABG or PTCA), stroke (including ischemic and hemorrhagic stroke);
8. Severe liver and kidney dysfunction (ALT three times greater than the upper limit of the normal and creatinine >132 μmol/l);
9. After the run-in period, fasting TG is <5.6mmol/L;
10. Pregnant or lactating women, and women of childbearing age who have not taken effective contraceptive measures;
11. Participation in any other clinical trials;
12. For any reason, the investigator may consider a subject inappropriate for participation in this study.

#### Withdrawal Criteria

1. An allergic reaction that is clearly associated with the study drug;
2. Adverse symptoms or signs and abnormal examination results occur that are clearly related to intake of the study drug;
3. Women who develop pregnancy during the study;
4. Subjects request to withdraw from the study;
5. Signs of severe or frequent hypoglycemia: hypoglycemic events that require help from others (e.g., increased physical activity or no meals), or weekly hypoglycemic events [plasma or hand-pricked blood glucose measurements ≤ 70 mg/dL ( ≤ 3.9 mmol/L); any episode with or without symptoms] occurring more than three times. Cardiovascular or cerebrovascular events that occur during the study including acute myocardial infarction, stroke, hospitalization for heart failure, etc.

### Intervention

#### Treatment

Study group: general lifestyle intervention + Jinlida granules (three times a day at a dosage of 6 g each time) drug consumed with boiled warm water;

Control group: general lifestyle intervention + placebo (three times a day at a dosage of 6 g each time); drug consumed with boiled warm water;

The research drugs are recommended to taken after meals. If the patient has an intolerable adverse event that considered as relevant to the study drug based on the investigator's consideration, the patient should discontinue the medication. Treatment duration is at least 2 years.

#### General Lifestyle Intervention

The Guidelines for the prevention and treatment of type 2 diabetes (2017 edition) (3) suggest that subjects with abnormal glucose metabolism should reduce their risk of diabetes through diet control and exercise. Regular follow-up and psychosocial support to ensure that lifestyle changes can persist for a long time are needed; as well as checking blood glucose regularly and paying close attention to other cardiovascular risk factors (such as smoking, hypertension, dyslipidemia, etc.) including giving appropriate intervention measures. The primary objectives of the intervention are as follows:

1. Maintaining a healthy weight: the weight loss goal for overweight/obese subjects is 5–10% of weight loss in 3–6 months. A lean patient should maintain an ideal body weight through a reasonable nutrition plan.
2. Providing a balanced diet to meet the physical demand for micronutrients.
3. Achieving and maintaining ideal blood glucose levels as well as reducing HbA1c levels.
4. Reducing risk factors for cardiovascular disease including control of dyslipidemia and high blood pressure.

In addition, dietary and exercise intervention as well as smoking cessation are important in demonstrating the value of metabolic syndrome control. All recommendations were recommended by the guidelines (3) and related details are also uploaded to the Supplementary Material.

Emphatically, in this study, most subjects come from the communities and hospitals. We designate the community workers or medical stafvies to manage the patients for lifestyle intervention and keeping detailed records in the designed form, in order to maintain two groups in balance.

### Study Procedure

Figure 1 shows the study flow and Table 1 shows the study time schedule. The study will include run-in period, random enrollment, and treatment period. The run-in period was 1 month, and treatment period is at least 2 years. All the subjects will receive oral and written information regarding the study. Written informed consent will be obtained by the doctors involved in the trial.

**Table 1:** Study flow chart.

**Research phase**	**Run-in**	**Random enrollment**	**Treatment period**													
Time	−1 Month	−7–0 days	1 M ± 7days	2 M ± 7	3 M ± 7	4 M ± 7	5M ± 7	6 M ± 7	7 M ± 7	8 M ± 7	9 M ± 7	10 M ± 7	11 M ± 7	12 M ± 7	Until the 48th month^#^	When an end event occurs
**Basic Information**																
Informed consent form	•															
General information and medical history		•														
Vital signs	•	•	•	•	•	•	•	•	•	•	•	•	•	•	•	•
**Treatment**																
Inclusion/exclusion criteria	•	•														
Assign random number		•														
Distributing research drugs		•			•			•			•			•	※	
Record combined medication		•			•			•			•			•	※	•
Drug recovery					•			•			•			•	※	•
**Safety indicators**																
Blood/urine routine		•						•						•	※	•
Liver and kidney function		•						•						•	※	•
12-lead ECG		•						•						•	※	•
Serum NO, E-1		•												•	※	•
Thyroid function		•														
NYHA classification		•														
Adverse event assessment	•	•	•	•	•	•	•	•	•	•	•	•	•	•	•	•
**Efficacy index**																
Fasting blood glucose (capillary)	•	•	•	•		•	•		•	•		•	•		※	
Two hours post-prandial blood glucose (capillary)	•	•	•	•		•	•		•	•		•	•		※	
OGTT test		•	⊚	⊚	•	⊚	⊚	•	⊚	⊚	•	⊚	⊚	•	※	•
Four blood lipids		•						•						•	※	•
Weight, BMI, waist circumference	•	•	•	•	•	•	•	•	•	•	•	•	•	•	•	•
Fasting insulin		•						•						•	※	•
Glycated hemoglobin		•						•						•	※	•
C-reactive protein		•						•						•	※	•
Urinary microalbumin		•						•						•	※	•
Carotid ultrasound		•												•	※	•
Brachial index		•												•	※	•

# *13th−48th month of follow-up, please continue the first 12 months of the visit; ⊚: If the blood glucose measured by the capillaries is diagnosed as diabetes, please perform the OGTT test. ※: Please check the 1st−12th months of follow-up at the end of the 13th to 48th month. •: Items to be performed during follow-up*.

### Screening Cycles

Subjects who are diagnosed with impaired glucose tolerance syndrome will be given a 1-month general lifestyle intervention.

This visit should include the following:

1. Signing informed consent;
2. Physical examination;
3. Detection of fasting blood glucose and 2-h postprandial blood glucose by capillaries;
4. Guidance for subjects on general lifestyle interventions.

### Intervention Cycles

Eligible subjects will be randomly assigned to receive general lifestyle intervention + Jinlida granules at a dose of 9 g or general lifestyle intervention + placebo, orally, three times daily. The run-in period is 1 month, and the intervention will last 24–48 months. Follow-up will last 24 months.

### Outcome Measure

#### Primary Outcomes

Incidence of type 2 diabetes.

#### Secondary Outcomes

1. Changes in the number of components of the metabolic syndrome;
2. Changes in single indicators (waist circumference, blood pressure, TG, HDL-C) in metabolic syndrome;
3. Carotid intima-media thickness (IMT);
4. Fasting insulin;
5. Ankle brachial index;
6. Glycated hemoglobin (HbA1c);
7. Endothelial function test: Serum NO, ET-1;
8. C-reactive protein;
9. Urinary microalbumin to creatinine ratio (ACR).

### Sample Size Calculation

According to “Lian et al. (23),” after 1 year of follow-up, the average annual incidence of diabetes in the placebo group was 29.32%. “Gao et al. (24)” demonstrated a cumulative incidence of diabetes in placebo group after 3 years of follow-up was 43.86%.

Our hypothesis assumes that the incidence of diabetes in the control group would be 40.0% after 2 years of intervention and the HR would be expected to be 0.75—the study drug is predicted to reduce the risk by 25%—employing α = 0.05, β = 0.20. The study group and the control group will be allocated in a 1:1 ratio. The study period will last 4 years, including 2 years of medication treatment and 2 years of follow-up. PASS16 software will be employed to calculate the sample size, each group should contain 395 subjects. Considering a 10% the violation rate and issues of inter-center case allocation, a total of 880 cases and evaluation of number of endpoint events 209:171 will be required to reach statistical significance.

### Randomization

Subjects who sign an informed consent form will receive a screening number consisting of two parts. The first two digits are the center number, and the last three digits are sequentially added according to the order in which the subjects are screened. Subjects will be randomized using a central randomized system. When the subjects meet the enrollment criteria, the clinical study coordinator will be required to enter the subject's general information (name, age, gender) into an interactive voice/network response system (IWRS/IVRS). There they will obtain a random number and a drug number for a corresponding visit. Drugs will be distributed randomized subjects according to the drug number.

### Blinding

The drug packaging shall be prepared by the blinding personnel of the statistical unit which is the unrelated the trial. According to the drug packaging number generated by the software and the corresponding intervention group, the drug packaging number of the research drug and the control drug shall be filled in (or pasted on) the label. The second-level blind bottom will be produced during on-the-spot blinding, which is reserved in sponsor unit.

This trial will use a central randomized system to dispense medications during the follow-up period, so the medications are packaged at follow-up intervals. The random number assigned to each subject will be unique, except that the package number of the drug received during different follow-up period will differ, but the corresponding treatment plan will remain consistent. All blinded personnel must sign the blinding record and make a document for the clinical trial. The drug package number and confirmation code information will be imported into the DAS for IWRS system after blinding, and are used when applying for random numbers, dispensing drugs, and unblinding online.

### Data Collection and Management

The investigator should ensure that the data is correctly, completely, clearly, and timely entered into the case report form based on the original observations of the subject. The auditor shall monitor whether the research is conducted in accordance with the research plan and confirm that whether all case report forms are completed correctly and consistent with the original data. If errors and omissions are made, the researcher shall be promptly corrected. The case report form after the inspection by the auditor needs to be transmitted to the data administrator of the clinical research in time. The data administrator should compile the data entry-program for data management. In order to ensure the accuracy of the data, two data entry personnel should independently perform double entry and proofreading. For questions in the case report form, the data administrator will generate a question-answer form and send an inquiry to the researcher through the clinical monitor. The researcher should answer the question as soon as possible, and the data administrator will modify the data according to the researcher's answer. If necessary, a question answer form can be issued again.

### Monitoring

An independent Data and Safety Monitoring Board will be set up prior to the start of the study. The DSMB will review data after the first participant, and then after the recruitment of 25, 50, and 75% of participants to check the study progress and all adverse events.

### Statistical Analysis

After the research trial is finalized, a statistical professional is responsible for developing a statistical analysis plan in consultation with the principle investigator. The statistical analysis software (SAS® 9.2 software) will be used.

#### Analysis Subjects

The research subjects will be divided into the following categories:

1. Full Analysis Set (FAS)
□ FAS refers to the data set obtained from all randomized subjects with the least amount and reasonable method of eliminating subjects. Exclusions usually include violations of important inclusion criteria; subjects do not receive treatment with the test medication; no observational data is obtained after randomization.
2. Per Protocol Set (PPS)
□ PPS is a subset of the full analysis set, and these subjects are more compliant with the trial. Subjects included in the PPS generally have the following characteristics: (1) the minimum exposure of the research drug in advance-completion, that is, the compliance with the drug is 80%; (2) the data of the primary outcomes in the test are available; (3) no major violation of the protocol.
3. Security data set (Safety Set, SS)
□ SS is a subject who received at least one-time treatment after randomization and has a safety assessment.

#### Statistical Analysis Method

□ All statistical tests will be performed using a two-sided test, and a *p* <0.05 (two-sided test) will be considered to be statistically significant results (except for special instructions).
□ Descriptive analysis: This data is described by the number of cases and the composition ratio. The measurement data are described by means of mean, standard deviation, maximum value, and minimum value. The non-normal distribution data are described by median, 25th and 75th quantiles. A comparison between the two groups of general conditions will be based on outcomes. Quantitative data will be compared between groups using group *t*-test or Wilcoxon rank sum test, classification data using chi-square test, or exact probability method, grade data used Wilcoxon rank sum test, or CMH test, as appropriate.

### Safety Assessment

The subjects will be described as the normal before treatment and the abnormal cases after treatment, rating the proportion of the number of cases. Adverse events will be described by the number of occurrences, the number of cases, and the incidence of adverse events. The incidence will be tested for significance between groups. At the same time, the specific performance and extent of all adverse events in each group of cases and their relationship with drugs should be described in detail.

### Current Status

Patients started enrolling in this trial on August 2019. As of May 2020, 182 patients were screened and 65 identified as candidates signed the informed consent. Currently, there is no subject completing the study. Relevant protocol modifications will be registered at http://www.chictr.org.cn. Trial registration number: ChiCTR1900023241

## Discussion

Jinlida is a Chinese patent medicine with significant hypoglycemic effect that has been used in China as part of T2DM interventions (3) and reduces the incidence of diabetes (23). Findings from a meta-analysis demonstrated Jinlida granules significantly reduced in fasting plasma glucose, 2-h post-load glucose, and HbA1c levels in patients with T2D (25). Pharmacologically, Jinlida has been reported to reduce intracellular lipid accumulation, enhance autophagy in NIT-1 pancreatic β-cells and increase expression of genes involved in mitochondrial function and fat oxidation (14, 19, 20). This randomized controlled trial will provide the first test of efficacy of Jinlida granules combination with lifestyle intervention. This work is innovative in several ways. First, although a handful of studies have been demonstrated that lifestyle intervention is an effective strategy to treat metabolic syndrome, delaying and inhibiting the progression of diabetes, many individuals are still progressing due to poor adherence. Further, due to impaired glucose tolerance (IGT) as the only reversible stage of type 2 diabetes and independent high risk factor for cardiocerebrovascular diseases and metabolism syndrome, it poses new challenges for metabolism syndrome patients management. This study incorporates a positive therapy framework, which offers a shift from traditional lifestyle intervention models toward a focus on enhancing well-being and accentuating strengths of Jinlida combination with lifestyle intervention. As such it may have appeal to a broad range of IGT in metabolism syndrome and provides a novel alternative to existing approaches.

At present clinical practice, Western medicine mainly focuses on more MS components rather than holistic treatment. Targeted drugs for treating metabolic syndrome have not yet been developed. Traditional Chinese medicine (TCM) emphasizes holistic health care. MS is conceptualized as a state of imbalance in human function by TCM, which change our understanding of the diseases from controlling of a single hypoglycemic, anti-hypertensive, and lipid-regulating to holistic regulation. TCM has certain advantages and great potential for the prevention and treatment of MS. However, its efficacy has not been objectively evaluated. Based on the unique advantages of Jinlida granules in clinical practice, our research team conducted a randomized controlled trial to evaluate the efficacy and safety of Jinlida granules in combination with metformin in the treatment of type 2 diabetes. This study showed that, based on dietary control, exercise therapy, and metformin intervention, Jinlida Granules could control glycosylated hemoglobin in type 2 diabetes mellitus better, at the same time it placed an advantage in regulating lipid metabolism disorder, reducing waist circumference and body weight of subjects. The results of these clinical research studies have elevated Jinlida granule treatment into the “Guidelines for the Prevention and Treatment of Type 2 Diabetes in China 2017” (25).

However, the previous clinical subjects in studies of Jinlida granules are mainly type 2 diabetes subjects. A clinical trial on IGT subjects in metabolism syndrome has not been performed. By examining the effect of multiple targets (improving insulin sensitivity index and insulin resistance, reducing glycosylated hemoglobin, regulating abnormal lipid metabolism, reducing body mass index) in Jinlida granules, our research team will implement a randomized, double-blind, placebo-controlled, and multi-center clinical trial to investigate the efficacy and safety of intervention with Jinlida granules in metabolic syndrome with abnormal glucose metabolism. If efficacious, Jinlida granules + lifestyle intervention (JGLI) should have high potential for implementation. We believe the potential public health impact of JGLI could be substantial if the results of this clinical trial show efficacy for abnormal glucose metabolism in metabolic syndrome.

Although randomized controlled trials are considered the gold-standard study design to investigate the effectiveness of treatment interventions, there might also be potential limitations for this study.

Because the long treatment period and rigorous lifestyle intervention may lead to poor patient compliance, which might greatly reduce the therapeutic effect. In addition, even in multicenter trial, measurement error from laboratory testing is inevitable duo to different medical devices in this multicenter trial. Importantly, there might be a handful of individual differences in the process of MS, possibly leading to different efficacy of the drug. More effort should be made to answer these questions in future studies.

## Conclusion

This study investigates the therapeutic potential of Jinlida granules by itself and as an add-on treatment in addition to general lifestyle intervention to improve MS patients with IGT. Being an oral treatment, it is less invasive than current therapies, and it has an adequate level of safety supported by its recommended use for type 2 diabetes. No clinical work supports its clinical use against MS patients with IGT, but preclinical evidences are ample and strong. If shown to be effective, it will have the potential to impact on reducing the development of type 2 diabetes and risks of ASCVD in MS.

## Dissemination

After completing the study, all data, including beneficial and adverse events, of trial intervention will be communicated at scientific meetings and published in indexed peer-reviewed journals. If shown to be effective, the therapy program will be made available to the general public in China.

## Ethics Statement

The trial protocol has been approved by the Ethics Committee of Hebei Yilin Hospital (approval number 2019LCKY-006).

## Author Contributions

DJ and LH contributed to the drafting of the manuscript. FL, TX, and ZJ revised the manuscript. All authors agreed with the integrity of the study and gave their approval.

## Conflict of Interest

The authors declare that the research was conducted in the absence of any commercial or financial relationships that could be construed as a potential conflict of interest.
