# Corrigendum: Basis and Design of a Randomized Clinical Trial to Evaluate the Effect of Jinlida Granules on Metabolic Syndrome in Patients With Abnormal Glucose Metabolism

**DOI:** 10.3389/fendo.2020.00649

**Published:** 2020-09-03

**Authors:** De Jin, Lili Hou, Shuolong Han, Liping Chang, Huailin Gao, Yiru Zhao, Shenghui Zhao, Xuedong An, Guangyao Song, Chunli Piao, Fengmei Lian, Tong Xiao-lin, Zhenhua Jia

**Affiliations:** ^1^Department of Endocrinology, Guang'anmen Hospital of China Academy of Chinese Medical Science, Beijing, China; ^2^Department of Cardiovascularology, Key Laboratory of State Administration of TCM (Cardio-Cerebral Vessel Collateral Disease), Shijiazhuang, China; ^3^Department of Cardiovascularology, National Key Laboratory of Collateral Disease Research and Innovative Chinese Medicine, Shijiazhuang, China; ^4^Department of Cardiovascularology, Key Disciplines of State Administration of TCM for Collateral Disease, Shijiazhuang, China; ^5^National Administration of Traditional Chinese Medicine (TCM) Regional TCM Diagnosis and Treatment Center (Cardiovascular Disease), Shijiazhuang, China; ^6^Department of Endocrinology, Heibei Yiling Hospital, Shijiazhuang, China; ^7^Guangzhou University of Traditional Chinese Medicine, Shenzhen Hospital, Guangzhou University of Chinese Medicine, Shenzhen, China

**Keywords:** Jinlida granules, Chinese medicine, metabolic syndrome, abnormal glucose metabolism, clinical trial

In the original article, there was an error in the “Inclusion Criteria” section. The authors wish to correct this to:

Inclusion Criteria

1. Subjects are between 18 and 70 years of age;
2. After the run-in period, subjects should meet the diagnostic criteria for metabolic syndrome (3, 6):

- Indispensable indicators:
  Abdominal obesity (central obesity): waist circumference in male ≥ 90 cm, female ≥ 85 cm;
- Other indicators:
  At least two of the following four indicators can diagnose metabolic syndrome:
  ① Hyperglycemia: fasting blood glucose (FBG) ≥ 6.1 or 2 h after glucose load (2HPG) ≥ 7.8 mmol/L and/or have been diagnosed as diabetes and received treatments.
  ② Hypertension: blood pressure ≥ 130/85 mmHg and/or have been confirmed to be hypertension and received related treatments.
  ③ Fasting TG ≥ 1.70 mmol/L.
  ④ Fasting HDL-C <1.04 mmol/L.

3. After the run-in period, subjects should meet the diagnostic criteria for impaired glucose tolerance:
(a) Impaired fasting glucose (IGT): FBG < 7.0 mmol/L and 2HPG: 7.8–11.1 mmol/L.

The authors apologize for this error and state that this does not change the scientific conclusions of the article in any way. The original article has been updated.
