# Supplementary material for: Basis and Design of a Randomized Clinical Trial to Evaluate the Effect of Jinlida Granules on Metabolic Syndrome in Patients With Abnormal Glucose Metabolism
Source: Front Endocrinol (Lausanne). 2020 Jun 25;11:415. doi: 10.3389/fendo.2020.00415 (PMC7330093; doi:10.3389/fendo.2020.00415)
Supplement: Supplementary file 2 [file Table_2.docx]

| **Research phase** | **Run-in** | **Random enrollment** | **Treatment period** | | | | | | | | | | | | | |
| --- | --- | --- | --- | --- | --- | --- | --- | --- | --- | --- | --- | --- | --- | --- | --- | --- |
| Time | -1 Month | -7-0 days | 1m ± 7 days | 2M±7 | 3M±7 | 4M ± 7 | 5M±7 | 6M±7 | 7m ± 7 | 8M±7 | 9M±7 | 10m ± 7 | 11M  ±7 | 12M  ±7 | Until the 48th month^#^ | When an end event occurs |
| **Basic Information** | | | | | | | | | | | | | | | | |
| Informed consent form | ● |  |  |  |  |  |  |  |  |  |  |  |  |  |  |  |
| General information and medical history |  | ● |  |  |  |  |  |  |  |  |  |  |  |  |  |  |
| Vital signs | ● | ● | ● | ● | ● | ● | ● | ● | ● | ● | ● | ● | ● | ● | ● | ● |
| **Treatment** | | | | | | | | | | | | | | | | |
| Inclusion/exclusion criteria | ● | ● |  |  |  |  |  |  |  |  |  |  |  |  |  |  |
| Assign random number |  | ● |  |  |  |  |  |  |  |  |  |  |  |  |  |  |
| Distributing research drugs |  | ● |  |  | ● |  |  | ● |  |  | ● |  |  | ● | ※ |  |
| Record combined medication |  | ● |  |  | ● |  |  | ● |  |  | ● |  |  | ● | ※ | ● |
| Drug recovery |  |  |  |  | ● |  |  | ● |  |  | ● |  |  | ● | ※ | ● |
| **Safety indicators** | | | | | | | | | | | | | | | | |
| Blood/urine routine |  | ● |  |  |  |  |  | ● |  |  |  |  |  | ● | ※ | ● |
| Liver and kidney function |  | ● |  |  |  |  |  | ● |  |  |  |  |  | ● | ※ | ● |
| 12-lead ECG |  | ● |  |  |  |  |  | ● |  |  |  |  |  | ● | ※ | ● |
| Serum NO, E-1 |  | ● |  |  |  |  |  |  |  |  |  |  |  | ● | ※ | ● |
| Thyroid function |  | ● |  |  |  |  |  |  |  |  |  |  |  |  |  |  |
| NYHA classification |  | ● |  |  |  |  |  |  |  |  |  |  |  |  |  |  |
| Adverse event assessment | ● | ● | ● | ● | ● | ● | ● | ● | ● | ● | ● | ● | ● | ● | ● | ● |
| **Efficacy index** | | | | | | | | | | | | | | | | |
| Fasting blood glucose (capillary) | ● | ● | ● | ● |  | ● | ● |  | ● | ● |  | ● | ● |  | ※ |  |
| Two hours postprandial blood glucose (capillary) | ● | ● | ● | ● |  | ● | ● |  | ● | ● |  | ● | ● |  | ※ |  |
| OGTT test |  | ● | ◎ | ◎ | ● | ◎ | ◎ | ● | ◎ | ◎ | ● | ◎ | ◎ | ● | ※ | ● |
| Four blood lipids |  | ● |  |  |  |  |  | ● |  |  |  |  |  | ● | ※ | ● |
| Weight, BMI,  waist circumference | ● | ● | ● | ● | ● | ● | ● | ● | ● | ● | ● | ● | ● | ● | ● | ● |
| Fasting insulin |  | ● |  |  |  |  |  | ● |  |  |  |  |  | ● | ※ | ● |
| Glycated hemoglobin |  | ● |  |  |  |  |  | ● |  |  |  |  |  | ● | ※ | ● |
| C-reactive protein |  | ● |  |  |  |  |  | ● |  |  |  |  |  | ● | ※ | ● |
| Urinary microalbumin |  | ● |  |  |  |  |  | ● |  |  |  |  |  | ● | ※ | ● |
| Carotid ultrasound |  | ● |  |  |  |  |  |  |  |  |  |  |  | ● | ※ | ● |
| Brachial index |  | ● |  |  |  |  |  |  |  |  |  |  |  | ● | ※ | ● |

**#: 13th to 48th month of follow-up, please continue the first 12 months of the visit; ◎: If the blood glucose measured by the capillaries is diagnosed as diabetes, please perform the OGTT test. ※: Please check the first to 12 months of follow-up at the end of the 13th to 48th month.**
